# Emergence of rare SARS-CoV-2 subvariant LF.7.3 obtained from Moroccan COVID-19 patients

**DOI:** 10.1128/mra.00911-25

**Published:** 2026-01-09

**Authors:** Hamza Ghammaz, Adil El Hamouchi, Nadia Touil, Hicham El Annaz, Marouane Melloul, Abdelhamid Barakat, Elmostafa El Fahime

**Affiliations:** 1 Molecular Biology and Functional Genomics Platform, National Centre for Scientific and Technical Research (CNRST) 204579, Rabat, Morocco; 2 Faculty of Medicine and Pharmacy, Genomic Centre for Human Pathologies (GENOPATH), Neuroscience and Neurogenetics Research Team, University Mohammed V 107736 , Rabat, Morocco; 3 Laboratory of Genomics and Human Genetics, Institut Pasteur du Maroc 290327, Casablanca, Morocco; 4 Department of Bacteriology, Mohammed V Military Teaching Hospital, Rabat, Morocco; 5 University Mohammed VI of Science and Health (UM6SS) 486625 , Casablanca, Morocco; 6 Genomics and Molecular Biology, Mohammed Vi Center for Research and Innovation (CM6RI), Rabat, Morocco; 7 Cell Culture Unit, Center of Virology, Infectious and Tropical Diseases, the Mohammed V Military Training Hospital 479569 , Rabat, Morocco; 8 Faculty of Sciences, Microbiology and Molecular Biology Team, Center of Plant and Microbial Biotechnology, Biodiversity, and Environment, Mohammed V University of Rabat 107736 , Rabat, Morocco; Queens College Department of Biology, Queens, New York, USA

**Keywords:** surveillance studies, virology, SARS-CoV-2, mutational studies, Morocco, genome analysis

## Abstract

We report the coding-complete genome sequence of a rare severe acute respiratory syndrome coronavirus 2 (SARS-CoV-2) subvariant (LF.7.3) identified in Morocco. Genomic analysis indicates that these strains belong to the LF.7.3 sublineage, a descendant of the JN.1 variant within the Omicron lineage.

## ANNOUNCEMENT

In late 2019, the novel coronavirus, severe acute respiratory syndrome coronavirus 2 (SARS-CoV-2), responsible for COVID-19 emerged in Wuhan, China and rapidly triggered a worldwide pandemic. Belonging to the Coronaviridae family and Betacoronavirus genus, the virus continues to evolve rapidly, giving rise to novel subvariants. We report the coding-complete sequence of a SARS-CoV-2 strain from patients in Morocco classified within the LF.7.3 sublineage of the Omicron variant.

A nasopharyngeal swab specimen was collected on 15 February 2024 in Rabat, Morocco from a SARS-CoV-2-positive individual using a sterile synthetic swab and stored in viral transport medium (VTM) at −80°C until processing. Total RNA was extracted using the QIAamp Viral RNA Mini Kit (Qiagen, Cat. No. 52906) following the manufacturer’s protocol.

Whole-genome amplification and library preparation were performed using the Illumina COVIDSeq Assay (Cat. No. 20059717) following the manufacturer’s instructions. Sequencing was performed on an Illumina NextSeq 2000, generating 34,851,114 paired-end reads (151 bp) with 99.34% read pairing. The genome assembly was performed using reference-guided assembly with minimap2 v. 2.30 for read alignment to reference NC_045512.2 and bcftools v. 1.22 for genome extraction. Default parameters were used for all software. The resulting assembly achieved 175,986× coverage of the 29,903 bp genome with 38.00% GC content. Sequence quality and lineage assignment were performed with Nextclade v3.13.2 (https://clades.nextstrain.org) (1) and phylogenetic analysis using UShER: Ultrafast Sample placement on Existing tRee v0.6.6 (2). Mutations were identified using GISAID’s CoVsurver web application (https://gisaid.org/database-features/covsurver-mutations-app/) (3).

The LF.7.3 subvariant identified in Morocco harbors 108 mutations (1.11% of its 9,699 amino acid sequence) and was classified as rare according to GISAID CoVsurver (Table 1). We identified multiple spike protein mutations of interest, including L455S, F456L, N501Y, E484K, F486P, P681R, G142D, H69del, Y144del, and the 12-nucleotide insertion (ins16MPLF), alongside a 68-nucleotide gap compared to the reference. These mutations enhance transmissibility and immune evasion (4). L455S and F456L are linked to reduced neutralization by XBB.1.5-targeted vaccines. N501Y, E484K, and F486P increase ACE2 receptor binding affinity, while P681R enhances S1/S2 cleavage. The NTD mutations and unique ins16MPLF insertion likely alter antibody binding sites.

**TABLE 1:** Amino acid substitutions in the Moroccan SARS-CoV-2 LF.7.3 strain

Gene	Mutation
*NSP1*	S135R, L177F
*NSP2*	A31D
*NSP3*	T24I, V238L, G489S, K1155R, N1708S, S1717L, A1892T
*NSP4*	L264F, T327I, T492I
*NSP5*	P132H
*NSP6*	V24F, S106del, G107del, F108del, R252K
*NSP9*	T35I
*NSP12*	P323L
*NSP13*	R392C
*NSP14*	I42V
*NSP15*	T112I
*Spike*	Ins16MPLF, T19I, R21T, T22N, L24del, P25del, P26del, A27S, S31P, S50L, Q54H, H69del, V70del, L121I, V127F, G142D, Y144del, F157S, R158G, K182R, R190S, V213G, L216F, H245N, A264D, I332V, G339H, R346I, K356T, S371F, S373P, S375F, T376A, R403K, D405N, R408S, K417N, N440K, K444R, V445H, G446S, N450D, L452W, L455S, F456L, N460K, S477N, T478K, N481K, V483del, E484K, F486P, Q498R, N501Y, Y505H, E554K, A570V, D614G, P621S, H655Y, N679K, P681R, L721I, N764K, D796Y, S939F, N969K, P1143L
*E*	T9I
*M*	D3H, Q19E, T30A, A63T, A104V
*NS3*	T223I
*NS7b*	F19L
*N*	P13L, E31del, R32del, S33del, R203K, G204R, Q229K, S413R

**Fig 1:**
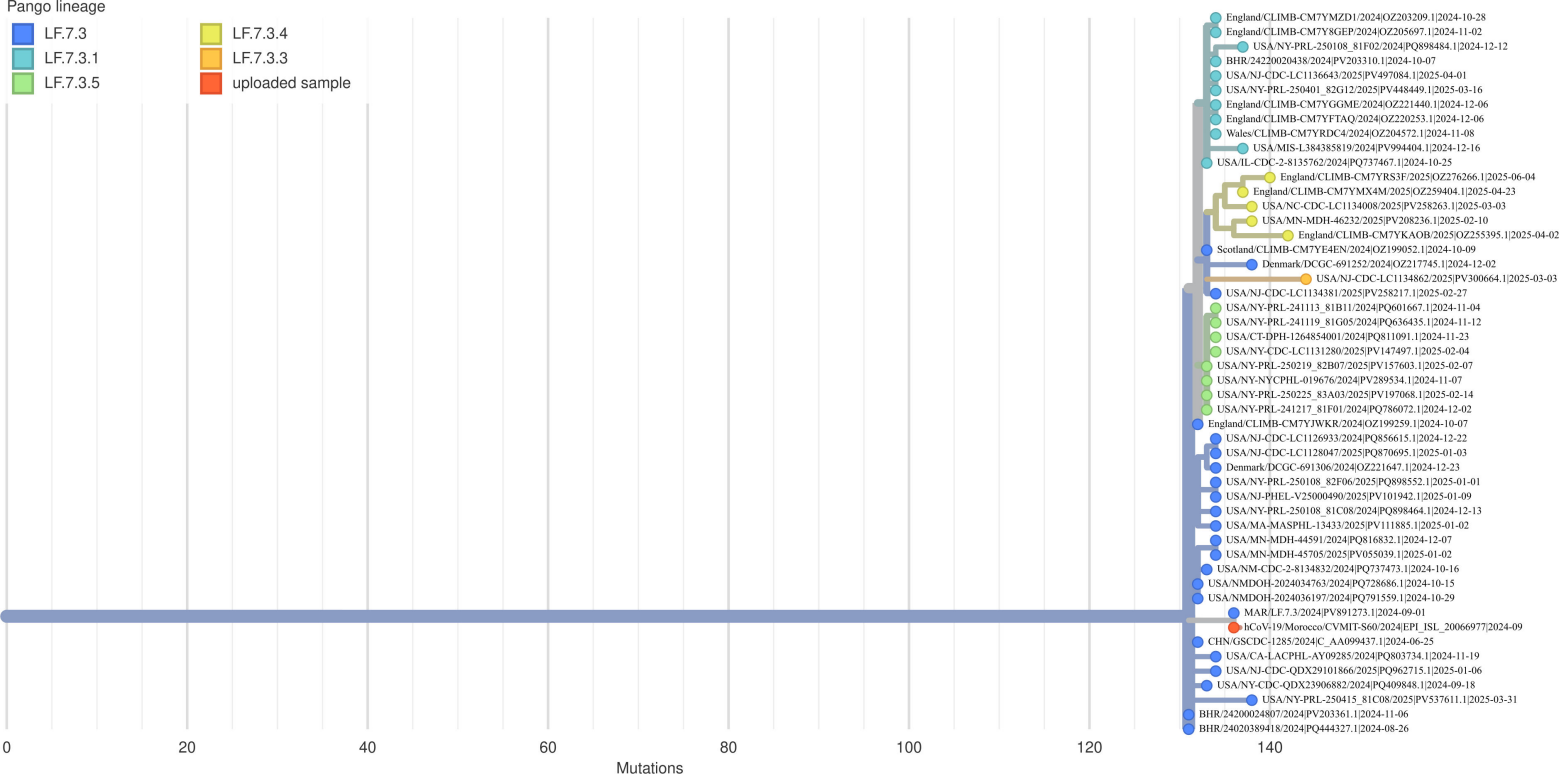
The phylogenetic tree generated using USHER depicts the evolutionary relationships among multiple samples based on Pango lineage assignments. Highlighted in red, the uploaded sample clusters within the LF.7.3.4 lineage, alongside related lineages, such as LF.7.3, LF.7.3.1, LF.7.3.2, and LF.7.3.5.

Phylogenetic analysis (Fig. 1) placed the Moroccan LF.7.3 genome within the broader LF.7.3 lineage, clustering specifically with LF.7.3.4 sublineage sequences. The tree topology confirms the Moroccan strain’s evolutionary relationship with contemporary circulating variants and demonstrates its placement within the dynamic Omicron variant ecosystem. LF.7.3 distinguishes itself from other Omicron subvariants through its non-recombinant origin and unique mutation profile. Current JN.1-targeted vaccines are expected to provide cross-protection against LF.7.3 due to antigenic similarity, although reduced neutralization efficiency is anticipated due to mutations L455S and F456L (5, 6).

The emergence of LF.7.3, including its detection in Morocco, underscores the critical importance of sustained genomic surveillance for tracking novel variants and assessing their potential impact on transmissibility and vaccine escape.

## Data Availability

The SARS-CoV-2 genome sequence of sample S60 has been submitted to the GISAID database under identifier EPI_ISL_20066977 and to NCBI GenBank with accession number PV891273.1. The raw reads are accessible via the BioProject database under SRA project number PRJNA1288432, with the specific raw read library for sample S60 listed as SRX29588391.
